# Extracellular pH affects the fluorescence lifetimes of metabolic co-factors

**DOI:** 10.1117/1.JBO.26.5.056502

**Published:** 2021-05-25

**Authors:** Rebecca Schmitz, Kelsey Tweed, Christine Walsh, Alex J. Walsh, Melissa C. Skala

**Affiliations:** a Morgridge Institute for Research, Madison, Wisconsin, United States; b University of Wisconsin-Madison, Department of Biomedical Engineering, Madison, Wisconsin, United States; c Texas A&M University, Department of Biomedical Engineering, College Station, Texas, United States

**Keywords:** autofluorescence, flavin adenine dinucleotide, NADH, fluorescence lifetime, pH, BT474, HeLa

## Abstract

**Significance:** Autofluorescence measurements of the metabolic cofactors NADH and flavin adenine dinucleotide (FAD) provide a label-free method to quantify cellular metabolism. However, the effect of extracellular pH on flavin lifetimes is currently unknown.

**Aim:** To quantify the relationship between extracellular pH and the fluorescence lifetimes of FAD, flavin mononucleotide (FMN), and reduced nicotinamide adenine dinucleotide (phosphate) [NAD(P)H].

**Approach:** Human breast cancer (BT474) and HeLa cells were placed in pH-adjusted media. Images of an intracellular pH indicator or endogenous fluorescence were acquired using two-photon fluorescence lifetime imaging. Fluorescence lifetimes of FAD and FMN in solutions were quantified over the same pH range.

**Results:** The relationship between intracellular and extracellular pH was linear in both cell lines. Between extracellular pH 4 to 9, FAD mean lifetimes increased with increasing pH. NAD(P)H mean lifetimes decreased with increasing pH between extracellular pH 5 to 9. The relationship between NAD(P)H lifetime and extracellular pH differed between the two cell lines. Fluorescence lifetimes of FAD, FAD-cholesterol oxidase, and FMN solutions decreased, showed no trend, and showed no trend, respectively, with increasing pH.

**Conclusions:** Changes in endogenous fluorescence lifetimes with extracellular pH are mostly due to indirect changes within the cell rather than direct pH quenching of the endogenous molecules.

## 1 Introduction

Two-photon fluorescence lifetime imaging (FLIM) of endogenous fluorophores can non-invasively monitor metabolic activity on a cellular level.^1^ Endogenous fluorophores related to metabolism include flavin adenine dinucleotide (FAD), reduced nicotinamide adenine dinucleotide (phosphate) (NAD(P)H), and flavin mononucleotide (FMN). FAD and FMN are fluorescent flavins, in which FAD is more prevalent in the cell.^2^^,^^3^ FAD and FMN are primarily localized in the mitochondria and bound to enzymes as a cofactor, although some proteins that use FAD or FMN as a cofactor are localized to other parts of the cell.^4^^,^^5^ Fluorescence spectra of NAD(P)H, FAD, and FMN have been previously published.^3^^,^^6^^,^^7^ The fluorescence spectra of FAD and FMN show substantial overlap, so FMN may contribute to FAD lifetime in FLIM measurements.^3^ NAD(P)H and FAD each have two distinct lifetimes due to free- and protein-bound states. For NAD(P)H, the short lifetime ($\tau_{1}$) corresponds to free, unbound NAD(P)H, and the long lifetime ($\tau_{2}$) corresponds to protein-bound NAD(P)H.^8^ Conversely, for FAD, free FAD has a long lifetime, and protein-bound FAD has a short lifetime.^9^

Given that FLIM of endogenous fluorescence is highly sensitive to cellular changes in metabolism, it has been used in many biomedical applications, including detection and treatment of cancer, tissue metabolism, and pathologies in the skin and eye.^10^^–^^17^ Fluorescence lifetimes are sensitive to a number of environmental factors, including pH, oxygen levels, and temperature.^10^^,^^11^^,^^18^^,^^19^ Extracellular pH is particularly relevant, as its effects on the internal pH of the cell may result in a suboptimal pH for metabolic enzymes to function or disrupt the proton gradient within mitochondria.^20^ Extracellular pH is typically maintained in interstitial fluid around pH 7.4.^21^ In some tissues, such as the GI system, the extracellular pH varies. In the intestinal tract, the pH may be as high as 8.0 or low as 4.0, and as low as 1.0 in the stomach.^22^ The extracellular pH is altered in common pathologies such as COPD, renal failure, and ischemia. The extracellular environment in tumors may also be more acidic (approximately pH 6.2 to 6.9) than healthy tissues.^23^^–^^25^ Studies in tumors have demonstrated that pH changes inside and outside the tumor are a major factor in promotion of tumor growth and metastasis.^23^^,^^26^

Several studies have investigated how pH affects the lifetime of various endogenous fluorophores. NAD(P)H mean lifetime in cells decreased with increased extracellular pH.^19^^,^^20^ Other studies focused on intracellular pH, using nigericin to equalize intracellular and extracellular pH across the cell membrane. These studies found NAD(P)H and FAD mean lifetimes decreased with increased intracellular pH.^27^^,^^28^ However, using nigericin/K+ to equalize pH across the membrane presents a confounding factor when used in conjunction with FLIM of metabolic cofactors. Nigericin decreases ATP concentrations, lowers the rate of protein synthesis, and affects the intracellular levels of other molecules, such as lactate.^29^^–^^31^ It is unclear whether the effects of nigericin are due to the presence of the molecule itself, or due to the changes in ion concentration and pH that result. At least, one study suggests that nigericin affects metabolites independently of its effects on ion concentrations.^29^ When nigericin/K+ equilibration is used with FLIM of NAD(P)H and FAD, molecules highly sensitive to metabolic changes, it may affect the results of such experiments. Since several studies have used nigericin to control intracellular pH, the effects of pH, independent of nigericin, on the fluorescence lifetimes of NAD(P)H and FAD remain ambiguous.

In addition, manipulation of extracellular pH often occurs in cell culture, as well as in other contexts, during FLIM experiments. For example, many cell media rely on ${\mathrm{CO}}_{2}$-dependent buffers to maintain media pH. If ${\mathrm{CO}}_{2}$ level is not maintained throughout the experiment, the buffer may no longer keep the extracellular pH consistent. Understanding the effects of extracellular pH on endogenous fluorescence lifetimes could improve the accuracy and reproducibility of *in vitro* and *in vivo* studies. Studies of extracellular pH effects on FAD lifetime are absent from the existing literature. Additionally, few studies have investigated pH effects on coenzyme lifetimes in more than one cell line, limiting our understanding of how extracellular pH may influence endogenous fluorescence lifetimes in different cell types.^19^

To further investigate the effects of extracellular pH on the fluorescence lifetimes of FAD, FMN, and NAD(P)H, human breast cancer (BT474) and HeLa cells were placed in pH adjusted media and imaged using two-photon FLIM. Additionally, 5-(and-6)-carboxy SNARF-1, a fluorescent intracellular pH indicator, was added to cells in a separate experiment to study the relationship between extracellular and intracellular pH changes. To investigate how these lifetime changes due to pH relate to the chemical properties of flavins, FAD (free and bound to cholesterol oxidase) and FMN solutions were prepared at varying pHs and imaged using FLIM.

## 2 Methods

### 2.1 FLIM of Cells and Solutions

BT474 cells (HER2 overexpressing human breast cancer cells) and HeLa cells were grown in Dubecco’s modified eagle medium (DMEM) supplemented with 10% fetal bovine serum and 1% penicillin: streptomycin. BT474 cells and HeLa cells were plated on glass-bottomed imaging dishes at $∼100,000\text{ cells}/{\mathrm{cm}}^{2}$ 24 h before imaging.

BT474 and HeLa cell samples were imaged in pH-adjusted DMEM media containing HEPES to maintain a consistent pH during imaging. The media pH was adjusted using 1.0 M NaOH and 1.0 M HCl to the desired pH for the sample, within a range of pH 4 to 9. This range of extracellular pH conditions includes much of the range of extracellular pH found in biological tissues.^21^^–^^25^ Cell samples were maintained in regular DMEM until 30 min before imaging, when the media were replaced with the pH-adjusted media. The control samples were imaged in non-pH-adjusted DMEM media containing HEPES. Dishes were imaged outside of incubation, so ${\mathrm{CO}}_{2}$ was present at atmospheric concentrations. These cells were imaged in media containing HEPES buffer. As HEPES buffer does not rely on ${\mathrm{CO}}_{2}$ for buffering capacity, this kept pH levels constant from the time of the initial media change through the end of imaging. Measurements of media pH performed after the experiment confirmed that the pH had not changed during imaging.

FAD salt hydrate (#F6625), cholesterol oxidase enzyme from *Streptomyces* sp. (#C8649), and FMN salt hydrate (#F2253) were purchased from Sigma Aldrich. FMN and FAD solutions were prepared in phosphate buffered saline, and cholesterol oxidase enzyme solutions were prepared in a 50-mM potassium phosphate buffer. All solutions were adjusted to a range of pH values, between pH 5 and 9, using 1.0 M HCl and 1.0 M NaOH. FMN was imaged at a concentration of 34  μmol/L and FAD at 340  μmol/L. The purchased cholesterol oxidase was non-covalently bound to FAD in a powdered form, so no additional FAD was added to these solutions.^32^ The solutions of cholesterol oxidase and FAD were dissolved in buffer and imaged at a concentration of 0.81  μmol/L.

All samples were imaged with a custom-built multiphoton fluorescence microscope (Ultima, Bruker) equipped with time correlated single photon counting electronics (SPC-150, Becker & Hickl GmbH, Berlin, Germany). A 40× (NA=1.15) water immersion objective was used and the imaging field of view was 270  μm×270  μm (256×256  pixels). The laser (Insight DS+, Spectra-Physics Inc., Santa Clara, CA, USA) was tuned to 750 nm for NAD(P)H excitation and 890 nm for FAD and/or FMN excitation. Fluorescence emission was isolated with 440/80  nm bandpass filter for NAD(P)H and a 540/80  nm bandpass filter for FAD and/or FMN.^3^^,^^6^^,^^7^ Fluorescence emission was detected with a H7422PA-40 GaAsP photomultiplier tube (Hamamatsu Corporation, Bridgewater, NJ, USA). Powers on the sample ranged from 3.0 to 5.0 mW (not including the SNARF measurements, which were measured at an average power of 0.3 mW). Control dishes were used for the purpose of checking for uniform excitation across the field of view. All images in this experiment were uniformly illuminated. A measured instrument response function (IRF) was also taken each day as a standard from the second harmonic generation of a urea crystal.

The decay curves for each pixel were obtained by binning each pixel with the eight surrounding pixels. The decay curves were then deconvolved using the measured IRF. In cells and cholesterol oxidase solutions, the resulting decay was fit to a two-component exponential decay function. This accounts for the two distinct lifetimes of NAD(P)H and FAD in the free and protein-bound state in cells.^8^^,^^9^ For NAD(P)H, $\alpha_{1}$ and $\tau_{1}$ correspond to free NAD(P)H; whereas for FAD, $\alpha_{1}$ and $\tau_{1}$ correspond to protein-bound FAD. We note that a two-component fit at 890 nm does not account for the possibility of three species (protein-bound FAD, free FAD, and FMN) and that FMN may contribute to the long lifetime at 890 nm.^33^ However, the use of a two-component fit enables comparisons to previous studies in cells that also use a two-component fit for the FAD channel^15^^,^^16^^,^^34^^–^^36^ and reduces the binning needed for reliable fits. For the cholesterol oxidase solutions, some of the FAD dissociated from the enzyme in the dissolution of the powder, so a two-exponential fit was used. The two-exponential fit function is

$$I(t)=\alpha_{1}e^{-\frac{t}{\tau_{1}}}+\alpha_{2}e^{-\frac{t}{\tau_{2}}}+C,$$

where I(t) is the light intensity at time t following the laser pulse, $\tau_{1}$ and $\tau_{2}$ represent the short- and long-fluorescence lifetimes of the fluorophore, and $\alpha_{1}$ and $\alpha_{2}$ represent the fractional contribution of each fluorescence lifetime.^9^^,^^12^ C accounts for the presence of background light. A mean lifetime ($\tau_{m}$) was also obtained for each pixel using the formula $\tau_{m}=\alpha_{1}\tau_{1}+\alpha_{2}\tau_{2}$. In FAD and FMN solution images, the decay at each pixel was fit to a one-component exponential decay function. These steps were carried out in SPCImage (Becker & Hickl).

For cell images, automated segmentation of cell cytoplasms was performed in CellProfiler.^37^ Nuclear regions of the cells were segmented by identifying pixels that were darker than the surrounding cytoplasm, but brighter than the background. The resulting round objects with diameter 10 to 40 pixels were saved as nuclei. Cells were identified by propagating out from the nuclei, using a threshold to prevent propagation into the background. Cell cytoplasm was defined as the cell region minus the nuclear region. 25 to 125 cells per sample were segmented. Values of fluorescence lifetime variables (FAD $\tau_{m}$, FAD $\tau_{1}$, FAD $\tau_{2}$, FAD $\alpha_{1}$, NAD(P)H $\tau_{m}$, NAD(P)H $\tau_{1}$, NAD(P)H $\tau_{2}$, NAD(P)H $\alpha_{1}$) as well as the optical redox ratio (defined as NAD(P)H intensity divided by the sum of NAD(P)H and FAD intensities) were calculated for each cell cytoplasm. The lifetimes of solution images were calculated for each pixel and averaged on an image level. Calculations were performed using MATLAB and R.

### 2.2 Intensity Imaging of pH Indicator SNARF-1

To study the relationship of extracellular pH and intracellular pH using an intracellular pH indicator, new samples of BT474 and HeLa cells were prepared and imaged separately from the FLIM experiments. Before imaging, a 10  μmol/L solution of 5-(and-6)-carboxy SNARF-1 (acetoxymethyl ester acetate), a fluorescent pH-sensitive compound (#C1272, Fisher Scientific), was prepared in serum-free DMEM. The cells were incubated in the 10  μmol/L SNARF-1 solution for 30 min. Following incubation, a pH-adjusted HEPES-containing media (pHs 4, 5, 6, 7, 8, and 9) replaced the SNARF-1 solution.

Nigericin equilibrizes intracellular and extracellular pH and was used for calibration purposes in the SNARF-1 calibration experiments only. Nigericin (#N1495, Fisher Scientific) at 10  μmol/L was prepared in buffer solutions (#P35379, Fisher Scientific) at pHs 4.5, 5.5, 6.5, 7.5, and 8.5. New samples of BT474 and HeLa cells were treated with the SNARF-1 solution for 30 min, then a nigericin-buffer solution was added to calibrate intracellular pH to the pH of the buffer.

Intensity images of the SNARF-1 fluorescence were collected using two-photon microscopy (as described above) for both experimental dishes (cells treated with pH-adjusted HEPES-containing DMEM media, no nigericin) and calibration dishes (cells treated with nigericin buffer solutions). The laser was tuned to 900 nm (average power: 0.3 mW) for SNARF-1 excitation. A 650/45  nm bandpass filter collected the pH-dependent SNARF-1 emission.^38^ A second low-pass filter collected all emission <575  nm to establish an isoemissive baseline for all samples. To create a calibration curve, the ratio of the intensity of the pH-dependent emission (650/45  nm bandpass) to baseline (575 nm low pass) was fit to a sigmoidal curve plotted against pH for each cell line treated with nigericin (calibration dishes).^39^ Intracellular pH values of the experimental dishes were found by calculating the SNARF1 intensity ratio (intensity of 650/45  nm bandpass divided by intensity of 575 nm low pass) and calculating pH from the sigmoidal fit of the calibration curve from the same cell line.

### 2.3 Statistics

Statistical analysis was performed in R. The linear correlation between pH and fluorescence variables in BT474 and HeLa cells was determined using the Pearson correlation coefficient. The statistical significance between Pearson correlations was determined using the Pearson R-to-z transformation. Statistical significance between pH conditions of the FAD, cholesterol oxidase, and FMN solutions was calculated using one-way ANOVA followed by Tukey’s HSD test.

## 3 Results

**Fig. 1:**
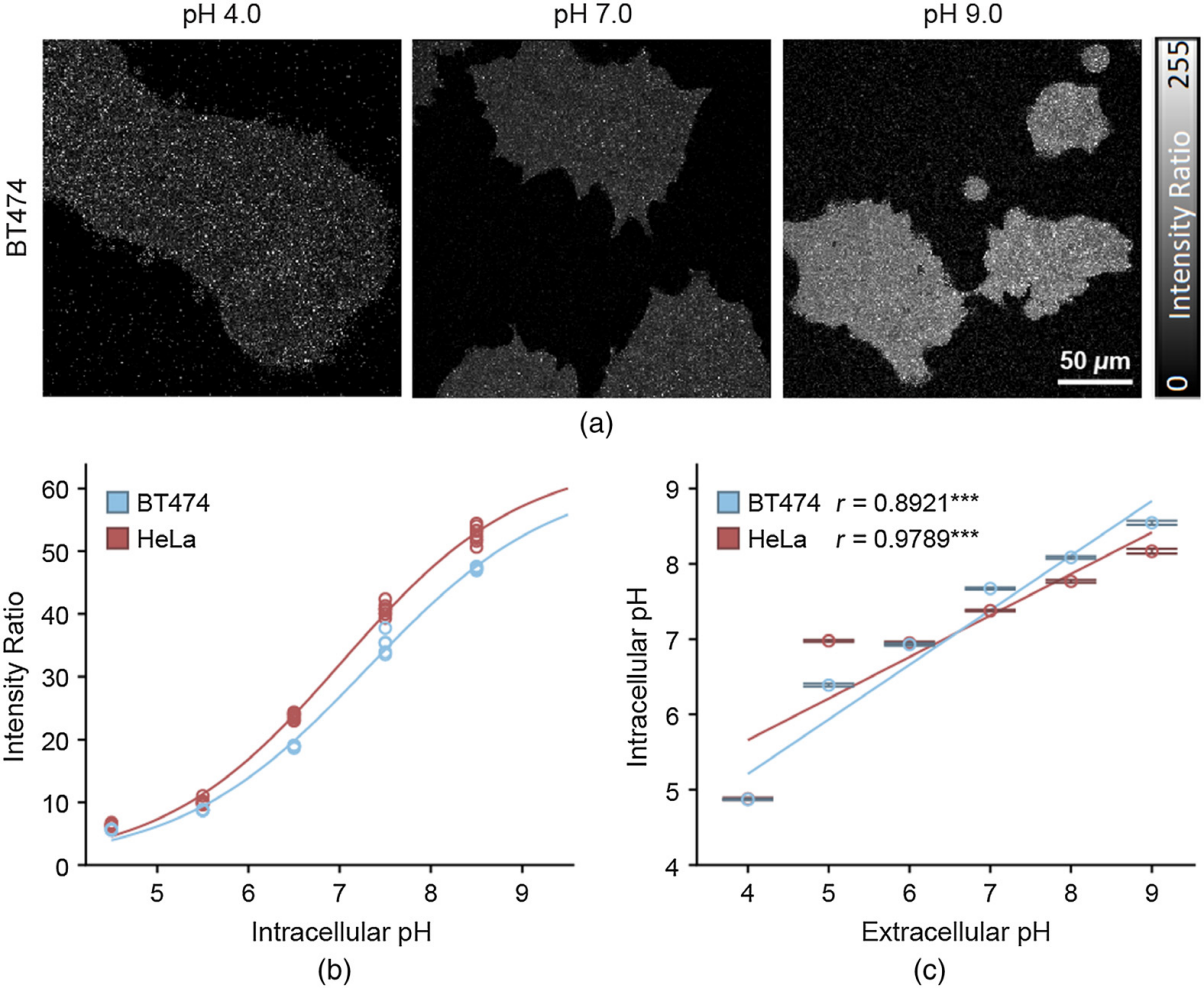
SNARF-1 measurement of intracellular pH. (a) Representative images of the intensity ratio of SNARF-1 fluorescent dye in BT474 cells. Intensity ratio is calculated on an image level and is equal to the intensity of the pH-dependent SNARF-1 intensity at 650/45  nm bandpass divided by the isoemissive SNARF-1 intensity at 575 nm low pass. (b) Calibration curve in BT474 (blue) and HeLa (red) cells, fit to a sigmoidal curve (n=65 images, n=25 images, respectively; each dot corresponds to one image). (c) Intracellular pH versus extracellular pH in BT474 (blue) and HeLa (red) cells, mean±95% confidence intervals (n=48 images, R=0.8785 and n=30 images, R=0.9789, respectively). ***P<0.001. R-values correspond to Pearson correlation coefficients.

Representative images of the SNARF1 intensity ratio in calibration condition (nigericin treated) HeLa cells demonstrated a qualitative increase in the intensity ratio as the pH increased from pH 4 to pH 9 [Fig. 1(a)]. This was further supported by the quantitative calibration data, showing a sigmoidal relationship between the intensity ratio of SNARF-1 and the intracellular pH in both cell lines [Figs. 1(b) and 1(c)]. Using these calibration curves to calculate the intracellular pH in the experimental condition (no nigericin), a strong positive correlation between extracellular pH and intracellular pH emerged, present in both HeLa and BT474 cells [Figs. 1(d) and 1(e)]. The Pearson correlation coefficient (R) for this relationship was 0.8921 in BT474 cells and 0.9789 in HeLa cells.

The SNARF-1 data demonstrated the existence of a strong positive correlation between the extracellular and intracellular pH in BT474 and HeLa cells. Therefore, altering environmental pH does affect the intracellular pH. Given the pH dependence of many enzymes and metabolic processes, this is especially relevant for studies of metabolism. The relationship between intracellular and extracellular pH characterized in this assay is consistent with other studies investigating the same relationship.^20^^,^^39^^,^^40^

**Fig. 2:**
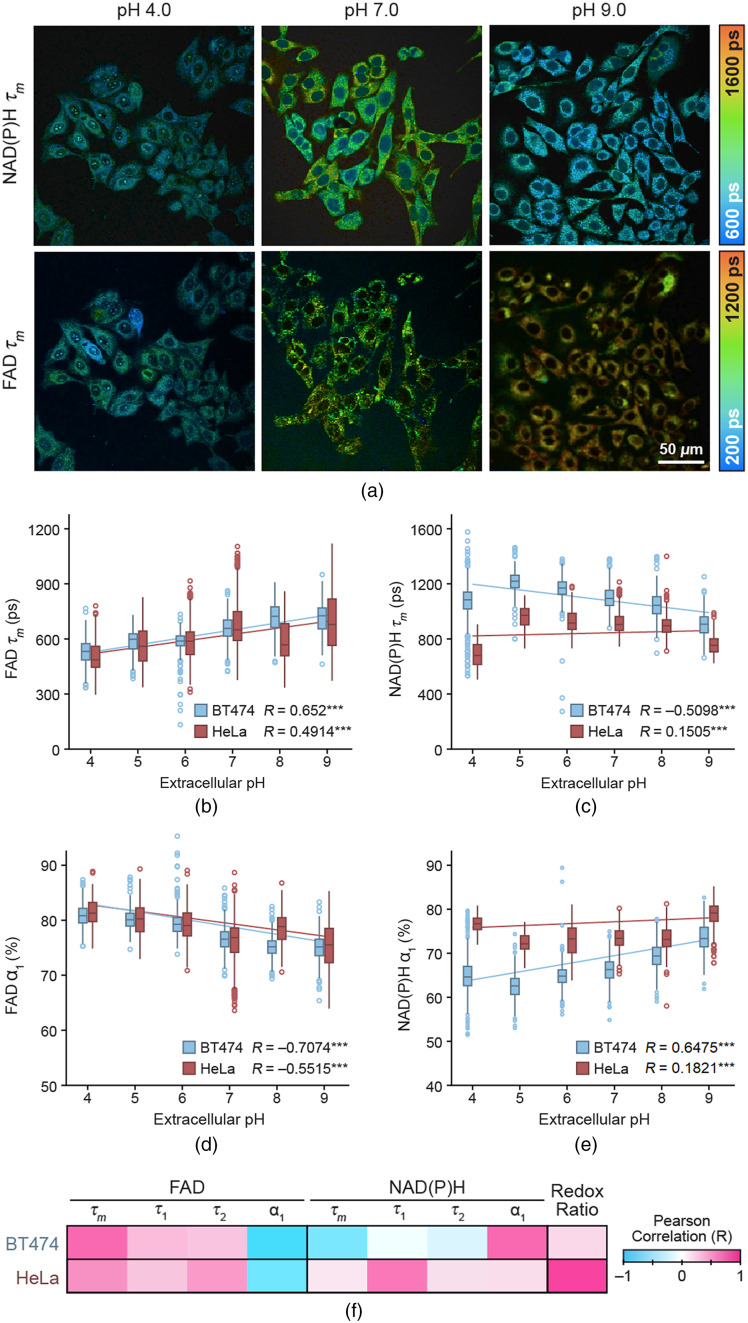
FLIM of BT474 and HeLa cells vary with extracellular pH. (a) Representative images of NAD(P)H $\tau_{m}$ and FAD $\tau_{m}$ in HeLa cells at pH 4.0, pH 7.0, and pH 9.0 in pseudocolor. (b) Box-and-whisker plot of FAD $\tau_{m}$ versus extracellular pH. Box is centered at median and reaches to first and third quartiles. Whiskers reach to 1.5 × interquartile range with dots as outliers beyond this range. (c) Box-and-whisker plot of NAD(P)H $\tau_{m}$ versus extracellular pH. (d) Box-and-whisker plot of FAD $\alpha_{1}$ versus extracellular pH. (e) Box-and-whisker plot of NAD(P)H $\alpha_{1}$ versus extracellular pH. Blue=BT474, red=HeLa. (f) Heatmap of Pearson correlations, measuring the linear correlation with pH for all metabolic parameters in both cell lines, for the extracellular pH range 4 to 9; all R-values are significant at p<0.05. n=4831 cells for BT474, n=3870 cells for HeLa. R values correspond to Pearson correlation coefficients. ***P<0.001.

Representative images of NAD(P)H $\tau_{m}$ and FAD $\tau_{m}$ in HeLa cells qualitatively demonstrated an increase in FAD $\tau_{m}$ with increasing extracellular pH, and higher NAD(P)H $\tau_{m}$ at pH 7 compared to pH 4 and pH 9 [Fig. 2(a)]. The FAD $\tau_{m}$ trends were also demonstrated in the quantitative data, which showed a moderate linear correlation between extracellular pH and FAD $\tau_{m}$ in both BT474 and HeLa cells (R=0.6520 and R=0.4914 in BT474 and HeLa cells, respectively) [Fig. 2(b)]. Conversely, the overall linear correlation between NAD(P)H $\tau_{m}$ and extracellular pH was weakly positive in HeLa cells and moderately negative in BT474 cells [Fig. 2(c)]. However, with the pH 4 group excluded, NAD(P)H $\tau_{m}$ showed a moderately negative linear correlation with extracellular pH (R=−0.7627 and R=−0.5655 in BT474 and HeLa cells, respectively). In addition, NAD(P)H $\tau_{m}$ increased from pH 4 to pH 5 in both BT474 and HeLa cells.

We then investigated which parameters contributed to the mean lifetime trends. In these results, $\alpha_{1}$ and $\tau_{1}$ correspond to free NAD(P)H and protein-bound FAD. Increases in FAD $\tau_{m}$ with increasing extracellular pH were driven primarily by a decrease in FAD $\alpha_{1}$ with increasing extracellular pH [Fig. 2(d)]. The trend in NAD(P)H $\tau_{m}$ appears to have been driven by different components between the two cell types. In BT474 cells, there was a moderate linear correlation in NAD(P)H $\alpha_{1}$ with increasing extracellular pH [R=0.6475, Fig. 2(e)]. These results indicate an increase in the amount of free FAD and NAD(P)H [due to increased FAD $\alpha_{2}$ and NAD(P)H $\alpha_{1}$] when extracellular pH increases in BT474 cells. The linear correlation between NAD(P)H $\tau_{m}$ and extracellular pH was weaker in HeLa cells [R=0.1821, Fig. 2(e)]. In contrast, NAD(P)H $\tau_{m}$ in HeLa cells was more strongly driven by decreased NAD(P)H $\tau_{1}$ with increasing extracellular pH [R=0.5960, Fig. 2(f)]. FAD $\tau_{1}$, FAD $\tau_{2}$, and NAD(P)H $\tau_{2}$ had moderate to weak linear correlations with extracellular pH in both cell lines [Fig. 2(f)]. The redox ratio in HeLa cells demonstrated a strong linear correlation with extracellular pH (R=0.8059), but the correlation was much weaker in BT474 cells [R=0.2091, Fig. 2(f)].

These data suggested that extracellular pH affects the lifetimes of both FAD and NAD(P)H in the cells. FAD $\tau_{m}$ linearly decreases between extracellular pH 4 to 9. NAD(P)H $\tau_{m}$ linearly decreases between extracellular pH 5 to 9, with pH 4 breaking this trend. Interestingly, the trends and the strength of correlations between fluorescence lifetime variables and extracellular pH were not the same in both cell lines, with differences particularly evident in the NAD(P)H lifetime variables and the optical redox ratio [Fig. 2(f)]. This suggests that the extracellular pH may not affect the metabolism of every cell line in the same way or to the same degree.

**Fig. 3:**
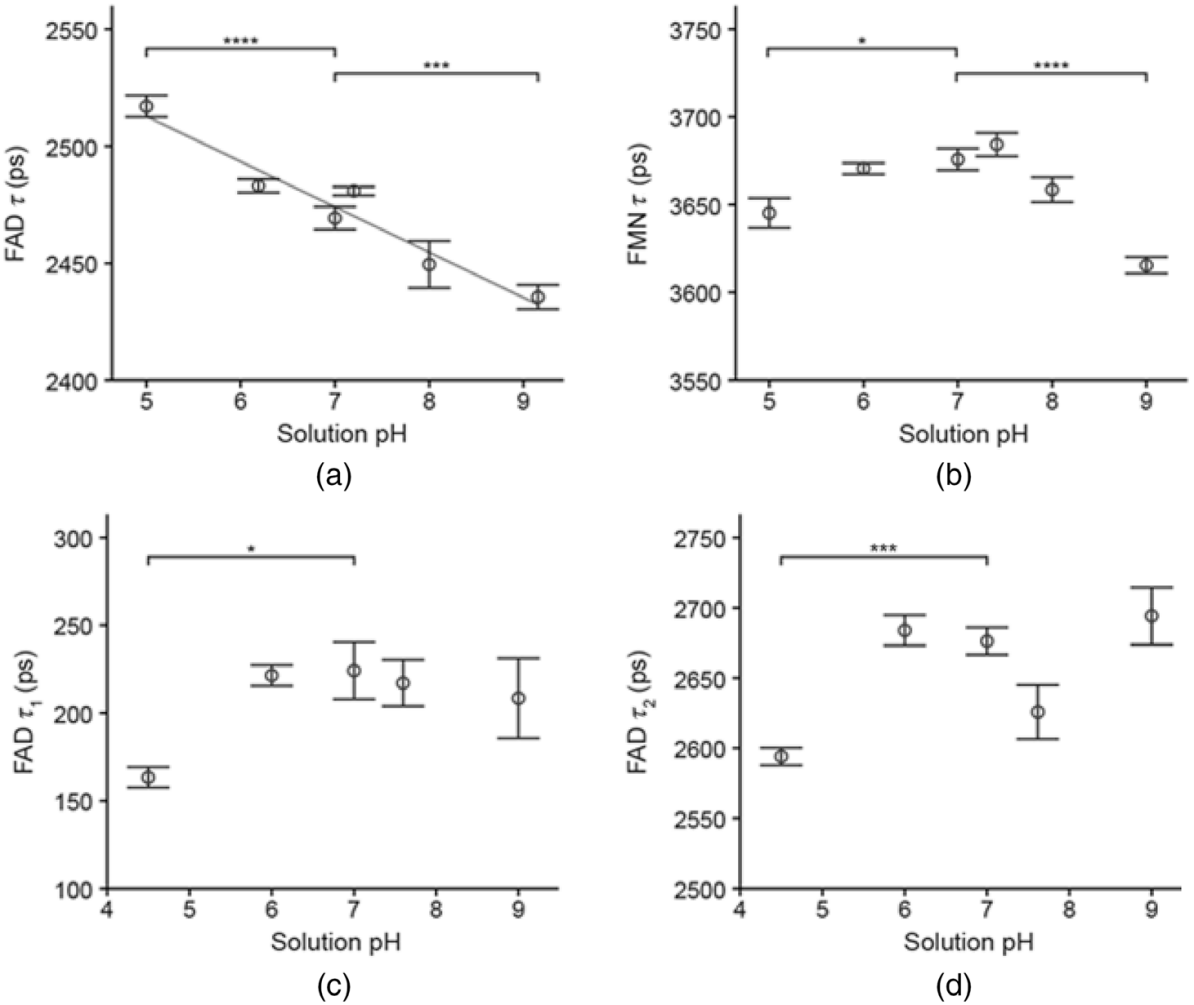
Fluorescence lifetimes of FAD and FMN solutions. (a) Fluorescence lifetimes of FAD solutions versus solution pH, mean±95% confidence intervals (linear fit is included with R=−0.8375, n=36 images). (b) Fluorescence lifetimes of FMN solutions versus solution pH, mean±95% confidence intervals (n=56 images). (c) Short fluorescence lifetime of FAD in solution with cholesterol oxidase versus solution pH, mean±95% confidence intervals (n=37 images). (d) Long fluorescence lifetime of FAD in solution with cholesterol oxidase versus solution pH, mean±95% confidence intervals (n=37 images). FAD and FMN solutions in (a) and (b) are fit to one component, FAD-cholesterol oxidase solutions in (c) and (d) are fit to two components. Data presented as mean±95% confidence intervals, p values compare group to control at pH 7.0. **** P<0.0001, *** P<0.001, * P<0.05.

In the FAD solutions [Fig. 3(a)], the magnitude of changes in FAD lifetime was small from pH 5 to 9, with a range of 2414 to 2534 ps. The FAD lifetime decreased with increasing solution pH [R=−0.8375, Fig. 3(a)], and the FAD lifetime in solution was significantly different from the pH 7 control at pH 5 and pH 9.2. FMN lifetime in solution [Fig. 3(b)] also exhibited only small changes, with a range of 3591 to 3704 ps over pH 5 to 9. Significant changes in FMN lifetime, compared to the control group at pH 7, occurred only in the pH 5 and pH 9 groups [Fig. 3(b)]. Cholesterol oxidase solutions containing FAD were fit to two components to account for both bound and free FAD. The proportion of free, unbound FAD in solution was 33.26%±5.19%. Both FAD $\tau_{1}$ (bound FAD) and FAD $\tau_{2}$ (free FAD) were significantly different from the control pH 7 only at pH 5 [Figs. 3(c) to 3(d)].

FAD $\tau_{2}$ trends in the cholesterol oxidase solutions differed from the FAD lifetime in solutions of FAD alone, but this difference may be attributed to the presence of cholesterol oxidase and its pH dependence in addition to pH-influenced changes in FAD itself. Cholesterol oxidase activity has been shown to vary with changes in pH.^41^ These changes in FAD and FMN lifetimes in solutions over pH 5 to 9 (Fig. 3) do not account for the observed changes *in vitro* in HeLa and BT474 cells (Fig. 2). This indicates that changes in flavin lifetimes observed *in vitro* are likely due to metabolic changes within the cell in response to pH rather than local changes in pH alone.

## 4 Conclusions

The results of this study demonstrate that alterations in extracellular pH result in changes to NAD(P)H and FAD fluorescence lifetimes. The strength of the correlations between endogenous fluorescence lifetime variables and extracellular pH were not conserved across cell lines, with differences being particularly evident in the NAD(P)H lifetime variables and the optical redox ratio. Studies of free FAD, bound FAD, and FMN solutions indicated that changes in cellular FAD lifetimes with pH are likely due to metabolic and/or enzymatic changes within the cell, rather than microenvironmental changes in pH around the fluorophore alone. This is consistent with changes in enzyme function that are known to occur with changes in cytosolic and mitochondrial pH.^20^ These results suggest that the relationship between extracellular pH and endogenous fluorescence lifetimes differ between cell lines, which could provide a method of cell phenotyping. This relationship between extracellular pH and endogenous fluorescence lifetime could also be applicable to quality control of cell conditions during FLIM imaging and to study metabolic changes in cells due to extracellular pH.
